# Supplementary material for: Mitigating stimulated Brillouin scattering in multimode fibers with focused output via wavefront shaping
Source: Nat Commun. 2023 Nov 13;14:7343. doi: 10.1038/s41467-023-42806-1 (PMC10643398; doi:10.1038/s41467-023-42806-1)
Supplement: Supplementary file 1 — Supplementary Information [file 41467_2023_42806_MOESM1_ESM.pdf]

Supplementary Information

Mitigating stimulated Brillouin scattering in multimode fibers with focused output via wavefront shaping

Chun-Wei Chen[1,+], Linh V. Nguyen[2,3,4,+], Kabish Wisal[5,+], Shuen Wei[2,+], Stephen C. Warren-Smith[2,3,4,*], Ori Henderson-Sapir[2,6], Erik P. Schartner[2], Peyman Ahmadi[7], Heike Ebendorff-Heidepriem[2], A. Douglas Stone[1,*], David J. Ottaway[2,6], and Hui Cao[1,*]

[1]Department of Applied Physics, Yale University, New Haven, 06520, CT, USA.
[2]Institute for Photonics and Advanced Sensing, School of Physics, Chemistry and Earth Sciences , The University of Adelaide, Adelaide, 5005, SA, Australia.
[3]Future Industries Institute, University of South Australia, Mawson Lakes, 5095, SA, Australia.
[4]Laser Physics and Photonics Devices Laboratoy, University of South Australia, Mawson Lakes, 5095, SA, Australia.
[5]Department of Physics, Yale University, New Haven, 06520, CT, USA.
[6]The Australian Research Council, Centre of Excellence for Gravitational Wave Discovery (OzGrav)
[7]Coherent, 1280 Blue Hills Ave., Bloomfield, 06002, CT, USA.
[+]These authors contributed equally to this work.
[*]Corresponding authors: Stephen.Warren-Smith@unisa.edu.au, douglas.stone@yale.edu, hui.cao@yale.edu

Abstract

This document provides supplementary information to "Mitigating stimulated Brillouin scattering in multimode fibers with focused output via wavefront shaping". In the first section we elaborate on the experimental setup and measurement procedure. In the second section, we present details of our theory and numerical simulations.

1 Experiment

1.1 Optical setup

We verify our scheme of SBS suppression by multimode excitation in two separate experiments. One is conducted with continuous waves (CW) at $\lambda = 1064$ nm, the other with 186-ns pulses at $\lambda = 1550$ nm.

Figure S1a depicts the first experimental setup. A fiber amplifier (research unit from Coherent Nufern), seeded by a CW fiber laser with a linewidth of 15 kHz (NP Photonics Rock 1μm), produces the linearly polarized signal (pump for Brillouin scattering). It is collimated by a lens and passes through an optical isolator to prevent the laser amplifier from being damaged by strong backscattered light from SBS. We use a zero-order half-wave plate (HWP) and a Glan-Taylor polarizer to control the signal power, a $4f$ system for beam expansion, and a phase-only spatial light modulator (SLM) for wavefront shaping. The signal beam is expanded to cover the active area of the SLM (Meadowlark HSP1920-500-1200-HSP8 with a water cooling system) to prevent damage at high power. The reflected light propagates through another $4f$ system with a pinhole at the focal plane to filter the zeroth-order diffraction from the SLM. It is then split by a non-polarizing beam splitter (BS), and the reflected light (10% of total power) is directed to a photodetector (PD) to monitor the input power. The transmitted light (90% of total power) is launched into a multimode fiber (MMF) by an objective lens (OL, μ-Spot LMH-20X-1064) for multimode excitation or a plano-convex lens (Thorlabs LA4725-1064) with a longer focal length (f = 75 mm) for fundamental-mode-only excitation.

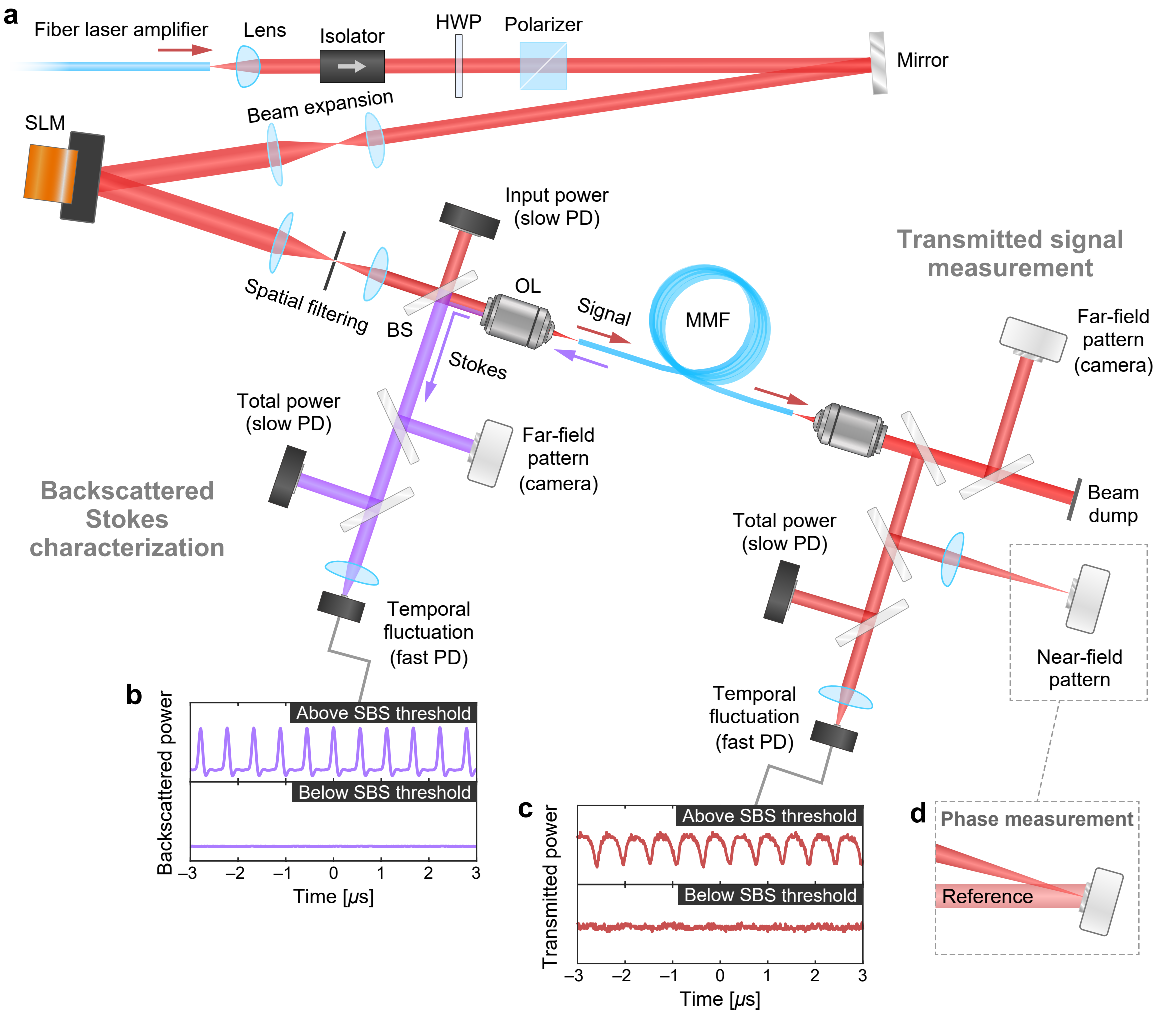

Fig. S1. Experimental setup for investigating SBS in a multimode fiber by wavefront shaping. **a,** Schematic showing the setup for wavefront shaping, input excitation, and output characterization. HWP: half-wave plate, SLM: phase-only spatial light modulator, BS: non-polarizing beam splitter with 10 % reflectivity, OL: objective lens with NA $\approx$ 0.3, PD: photodetector. The slow PD has a response time of $\sim$ms, and the fast PD of $\sim$10 ns. **b,c,** Measured time traces of backscattered (**b**) and transmitted (**c**) powers from a 50-meter-long MMF under CW excitation, above (upper) and below (lower) SBS threshold. SBS manifests as micro-second-scale pulsations of backward Stokes and forward signal. **d**, Off-axis holographic setup for phase measurement.

The MMF (Coherent Nufern FUD-3607) is 50 meters long and loosely coiled on an optical table without active cooling and mechanical isolation. The fiber core is germanium-doped, 20 μm in diameter, and has a numerical aperture (NA) of $\sim$0.3. The number of guided modes at the signal wavelength λ = 1064 nm is 80 per polarization. The transmitted light from the fiber is collected by an objective identical to that at the fiber input, and divided by beam splitters to several beams for simultaneous measurement of near-field and far-field intensity patterns, total power, and its temporal fluctuation. The near- and far-field patterns are captured by near-infrared cameras (Allied Vision Mako GM-419B-NIR and Xenics Xeva-1.7-320 TE3). The time-integrated power is measured by a slow PD (Newport 818-SL), while the temporal fluctuation on the sub-μs scale is recorded by a fast PD (Thorlabs DET10A or PDA20CS). Similarly, at the fiber input end, we measure the far-field intensity pattern of backscattered light, its time-integrated power and temporal fluctuation.

In addition to the output intensity profile, we also measure the phase pattern of the near field at the fiber distal end [Fig. 5b in the main text] in an off-axis holographic setup shown schematically in Fig. S1d. A fraction of the CW seed laser with a flat phasefront serves as a reference beam, and interferes with the transmitted light from the MMF. The two beams are incident onto a camera at

different angles, and their interference pattern is recorded by the camera. From it, we extract the phase pattern of the transmitted field. Figure 5b of the main text shows the measured phase across the focal spot near the fiber output end is flat, confirming diffraction-limited focusing through the MMF by shaping the input wavefront.

In the second experiment, we use a pulsed fiber laser (KEOPSYS, PEFL-E07-LP-040-200-010-W00-G3-T1-ET1-PE30D-CIRFA) that produces 186-ns pulses at a repetition rate of 10 kHz. The optical setup is similar to that of the first experiment with a CW laser. The SLM is Santec SLM-200, and an aspheric lens (Thorlabs C220TMD-C) is employed to couple light into the fiber. A quarter-wave plate (QWP) is inserted in the optical path between the SLM and the objective lens to convert the polarization state of light from linear to circular. A 50-meter-long MMF of the same core diameter and NA is used. It supports 37 modes per polarization at $\lambda = 1550$ nm. The time-integrated power of the transmitted pulses is measured by a power meter (Thorlabs S146C) with response time < 1 μs.

1.2 Characterization of SBS

The SBS threshold is determined from the dependence of the transmitted power on the input power. As shown in the upper panel of Fig. 2j in the main text, the time-integrated transmitted power first increases linearly with the input power, and then saturates at a certain power, which is set as the SBS threshold. The horizontal axis of Fig. 2j is the transmitted power in the absence of SBS, which is obtained from the input power and the MMF transmittance for a given modal content (determined by the launching condition of incident light). The SBS threshold is confirmed by the power variation of backscattered light [Fig. S2]. Below the threshold, the backscattered power is dominated by Fresnel reflection from the proximal fiber facet and Rayleigh scattering in the fiber, and it increases linearly with the input power. Once the transmitted power starts to level off, the Stokes power due to SBS becomes comparable to the power of linear backscattering, causing the total backscattered power to surge. To extract the Stokes power from the total power, we first perform a linear fitting of the total reflected power below the SBS threshold where the Stokes power is negligible, then extrapolate the linear fit to above the SBS threshold to obtain the power of linear reflection and backscattering. Next we subtract the linear power from the total reflected power and a small DC noise from the photodiode to obtain the Stokes power. Because of measurement noise, some values in the lower panel of Fig. 2j are negative, but close to zero, below the SBS threshold.

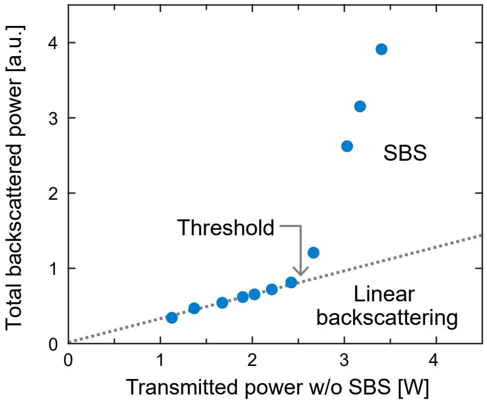

Fig. S2. Linear and nonlinear contributions to total reflected power. Experimentally measured, time-integrated power of light reflected from the multimode fiber as a function of the expected transmitted power in the absence of SBS. At low input power, Rayleigh backscattering in the fiber and Fresnel reflection from the fiber facet dominate over SBS, leading to a linear increase of total reflected power. Above a power threshold, the total reflected power surges, as SBS becomes dominant. Dotted line represents a linear fit of the total reflected power below the threshold. It is then extrapolated above the threshold to obtain the linear power, which is subsequently subtracted from the total power to obtain the Stokes power. The experimental data are taken for multimode excitation with on-axis input focusing.

At the onset of SBS, both transmitted signal and reflected Stokes exhibit micro-second-scale pulsations under CW excitation. Figure S1b,c shows periodic spikes of Stokes power and synchronized dips in transmitted signal power above the SBS threshold. The modulation period is about 0.5 μs, corresponding to the round-trip time of light propagation in the fiber [1, 2].

1.3 Single-mode vs. multimode excitation

Excitation of only the fundamental mode (FM) is achieved by using a plano-convex lens of NA ≈ 0.06 to focus the input light to the fiber proximal facet at normal incidence. The focal spot size is roughly equal to the fundamental-mode field diameter of the MMF. Under such excitation, the transmitted light from the MMF shows a single spot at the center of the far field, as expected for the FM [Fig. 2c in the main text]. The corresponding near-field intensity distribution is relatively smooth across the fiber core with low-contrast modulations from a small amount of higher-order modes (HOM), due to imperfect FM excitation at the input and/or linear mode coupling in the fiber [Fig. 2b in the main text]. Above the SBS threshold of 1.8 W, the backward Stokes beam profile is identical to that of the transmitted signal, indicating that SBS occurs predominantly in the FM.

To excite multiple modes in the fiber, we replace the lens with an objective lens of NA ≈ 0.3 (close to the NA of the MMF core). The focal spot on the proximal fiber facet is $\sim$3.4 μm, much smaller than the input beam size for FM-only excitation. In Figs. 2 and 3a of the main text, we shift the focus transversely across the fiber facet by displaying a linear phase ramp on the SLM. We gradually vary the ramping magnitude and orientation to control the deflection angle and direction, respectively. We measure the SBS thresholds for multiple focal spots with same distance d_{in} to the fiber axis but varying azimuthal angle. The averaged threshold increases with d_{in}.

1.4 SLM phase modulation

To obtain the data in Fig. 4 of the main text, we use the SLM to imprint a random phase pattern on the input beam to the MMF. The SLM is placed at the conjugate plane of the proximal fiber facet. The active area of the SLM (Meadowlark HSP1920-500-1200-HSP8) is 10.7×17.6 mm^2, consisting of 1152×1920 pixels. We group 144×144 SLM pixels into a macropixel. The dimension of one macropixel determines the lateral beam size at the fiber proximal facet. The phase of each macropixel is varied independently between 0 and 2π. Each SLM phase pattern comprises $\sim 8 \times 8$ macropixels over an area covered by the incident Gaussian beam. Reducing the macropixel size would increase the number of macropixels and thus the degree of control of input wavefront, but at the cost of diffraction loss from the abrupt change of phase from one macropixel to the next.

To further enhance the SBS threshold, we optimize the SLM phase pattern. Starting from a random phase modulation, we arbitrarily select a macropixel, scan its phase from 0 to 2π with a step of $\pi/10$, and evaluate the objective function, e.g., difference between the transmitted signal power P_{t} and the backward Stokes power P_{r}, for each phase value. After the scan, the phase of this macropixel is set to the value corresponding to the highest objective function. We continue to optimize another macropixel until all the phases of macropixels are optimized. The SBS threshold is increased after one round of optimization. We then iterate this process by starting another round of optimization. The SBS threshold usually saturates after three iterations with the same objective function, indicating that the optimization has converged to a local maximum of the SBS threshold. To escape from the local maximum, we then change the objective function to, e.g., P_{t}, and the threshold enhancement may rise slightly after one to two rounds of optimization. Figure S3 demonstrates one example of the threshold-optimization experiment with the pulsed laser at 1550 nm. This experiment begins with a random phase pattern of 15×15 macropixels and reiterates the pixel-by-pixel optimization with two different objective functions applied successively ($P_{\text{t}} - P_{\text{r}}$ and P_{t}). After 5 rounds of optimization of all macropixels, the threshold enhancement saturates at $3\times$ the threshold for FM-only excitation. Since the phase space is highly nonlinear, different starting phase patterns, search trajectories of macropixels, and objective functions have led to different optimized phase patterns with different SBS thresholds.

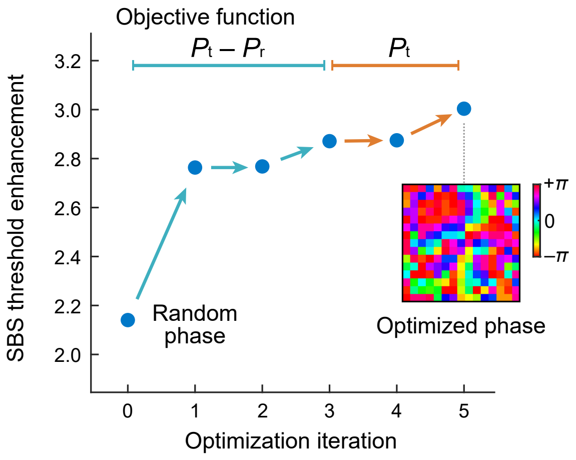

Fig. S3. SLM phase optimization for SBS threshold enhancement. One example of threshold-optimization experiment performed with the pulsed laser at $\lambda = 1550$ nm. Starting with a random phase pattern on the SLM, the SBS threshold enhancement over FM-only excitation is raised from 2.1 to 2.9 after three rounds of phase optimization over all (15×15) macropixels with objective function $P_\mathrm{t} - P_\mathrm{r}$, and further increases to 3.0 by switching the objective function to P_t for another two rounds of optimization. Inset shows the final SLM phase pattern of 15×15 macropixels.

As shown in Fig. 5 of the main text, we also search for the SLM phase pattern for focusing through a MMF. In order to excite as many fiber modes as possible, the axial distance from the focal spot to the distal fiber facet is less than $R/\tan\theta$, where R is the fiber core radius, and $\sin\theta$ is equal to the fiber core NA. We monitor the intensity distribution on a focal plane with a CMOS camera (Allied Vision Mako GM-419B-NIR) and select the position of focus within the field of view of the MMF. Output intensity at the selected location of focus is used as the objective function for optimizing the input phase pattern. The optimization process begins with a flat phase pattern. We select the macropixel located at the center of the incident Gaussian beam, where the intensity is maximal. We scan the phase of this macropixel from 0 to 2π and find the value at which the power at the focus is maximal. More specifically, we measure the power in the target area for 4–6 phases (ϕ) and fit the data with $\cos(\phi - \phi_0) + \text{const.}$, where ϕ_0 is the optimal phase value of the macropixel for focusing [3, 4]. After setting its phase to ϕ_0, we repeat the optimization process for a neighboring macropixel. We progress in a spiral-out trajectory to optimize all macropixels. Due to the smooth variation of the optimized phase over neighboring macropixels, the diffraction loss is small, allowing us to reduce the macropixel width to half of that for random phase modulation. The power at focus typically saturates after one or two rounds of optimization, and the optimized SLM pattern is a smooth phase modulation with $\sim 16 \times 16$ macropixels, as displayed in Fig. S4. We also note that other optimization approaches, e.g. the genetic algorithm described in ref. [5], can be adopted to find the SLM pattern for output focusing, but they often require fine tuning of the optimization parameters to achieve high focusing efficiency.

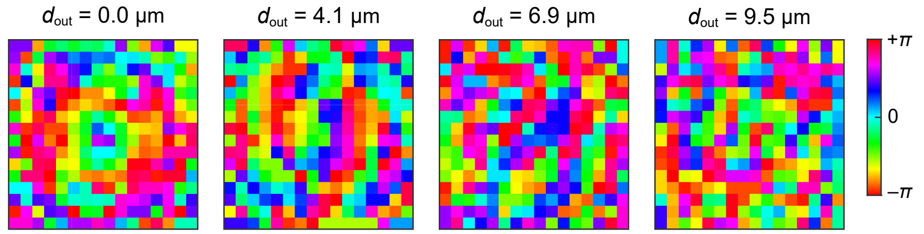

Fig. S4. Input phase modulation for output focusing. Optimized SLM phase patterns of 16×16 macropixels for focusing at different distances to the fiber axis: $d_\mathrm{out} = 0.0$, 4.1, 6.9, and 9.5 μm (left to right), showing smooth phase variations over neighboring macropixels. Input light is from a narrowband CW laser at $\lambda = 1064$ nm.

Thus far, we have used two types of objective function for optimizations. Functions such as $P_t - P_r$ or simply P_t are applied to achieve high SBS thresholds, but the output beam is highly speckled. Functions such as focal spot power (transmitted power in a target area of interest) or focusing efficiency (ratio of focal spot power over total transmitted power) are used to focus the output light to a single diffraction-limited spot. In this case, although the optimization for focusing also increases the SBS threshold due to multimode excitation, the threshold value is lower than what are achieved with threshold-only optimization. To find an optimal phase pattern that leads to a high SBS threshold while trying to concentrate as much power in the focal area as possible, objective functions such as "transmitted power in the target area minus backward Stokes power" can be used. We find experimentally (with the pulsed laser at $\lambda = 1550$ nm) that, compared to an objective function for simply maximizing the focusing efficiency, a higher power in the focal area at the SBS threshold can be obtained by applying such an objective function. For on-axis focusing, the threshold power becomes $\sim$50% higher, but the focusing efficiency is reduced by $\sim$8%. In turn, the SBS-limited maximum power in the focal spot is increased by $\sim$40%. The optimization also works for off-axis focusing but is less effective. For instance, at $d_{\text{out}} \approx 6$ μm, the threshold peak power is increased to from 64 W to 70 W, and the focusing efficiency drops from 0.63 to 0.58. However, the maximum focal power at the SBS threshold is only increased by $\sim$1%. This can be attributed to the fact that the SBS threshold is already high for off-axis focusing with the focusing-only optimization (as can be seen in Fig. 5c), thus the power-scaling improvement is relatively small.

1.5 Transmitted light linewidth

In this subsection, we provide experimental verification that the multimode excitation by input wavefront shaping does not cause any spectral broadening of the transmitted light. Using the self-heterodyne detection method [6], we record the spectra of the pulsed laser (pulse duration = 186 ns, $\lambda = 1550$ nm) before and after propagating through the multimode fiber. The results are presented in Fig. S5. The measured linewidth of the input light (blue trace) is about 2.4 MHz, as expected for transform-limited pulses of 186 ns. Figure S5 shows the spectra of the transmitted light measured under two launching conditions: (i) random input wavefront for multimode excitation, which leads to a speckled output (green trace), (ii) optimized input wavefront for output focusing (purple trace) which also results in multimode excitation. In both cases, the SBS thresholds are higher than that of FM-only excitation. The spectra in Fig. S5 are taken at the transmitted power of 50% higher than the FM-only SBS threshold, but still below the multimode SBS threshold. These results confirm that the transmitted linewidth (gray dashed arrow) remains 2.4 MHz, identical to the input linewidth.

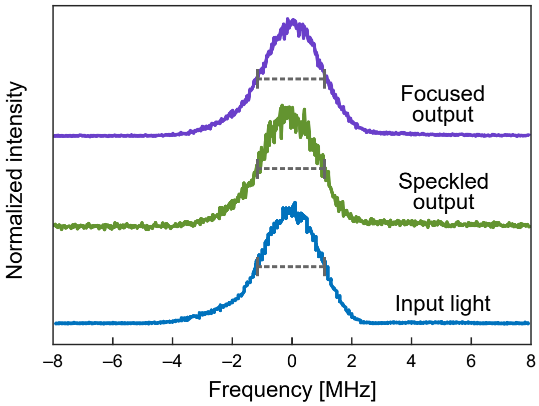

Fig. S5. Transmitted spectra under multimode excitation. Measured spectra of input laser pulse of duration 186 ns at $\lambda = 1550$ nm (blue) and of transmitted light through a 50-meter-long multimode fiber under multimode excitation that produces speckled output (green) and focused output (purple). The transmitted spectra are taken at power 1.5× the SBS threshold for fundamental-mode-only excitation, but below that for the multimode excitation. Transmitted light linewidth (dashed arrow) remains 2.4 MHz, which is identical to the input linewidth, confirming multimode excitation does not cause any spectral broadening. Traces are offset vertically for ease of viewing.

2 Theory and simulation

2.1 Multimode excitation by focused input

We simulate input focusing to different positions of the fiber core to calculate the signal mode contents $\{P_l\}$ and predict the corresponding SBS thresholds using our multimode SBS theory. Figure S6a–c shows the numerical estimation of the excited mode contents for (a) FM-only excitation, (b) on-axis focusing and (c) off-axis focusing for multimode excitations, demonstrated experimentally in Fig. 2 of the main text. The effective number of excited modes is defined as $M_{\text{eff}} = (\sum_l P_l)^2 / \sum_l (P_l{}^2)$. On-axis focusing excites only a few radial HOMs, and $M_{\text{eff}} \approx 4$ [Fig. S6b]. Off-axis focusing excites additional HOMs with non-zero azimuthal index [Fig. S6c], and M_{eff} increases monotonically with the distance d_{in} between the focal spot and the fiber axis [Fig. S6d]. Figure S3e shows the predicted SBS threshold enhancement over FM-only excitation as a function of M_{eff}. From $d_{\text{in}} = 0$ to $8\ \mu$m, M_{eff} increases gradually from 4 to 30, leading to an increase in the SBS threshold enhancement from 1.4 to 2.3 [Fig. S6e]. Moving beyond $d_{\text{in}} = 8\ \mu$m, M_{eff} rapidly rises from 30 to 85 at the fiber core edge ($d_{\text{in}} = 10\ \mu$m), and the final threshold enhancement of $\sim$3 is predicted [Fig. S6d,e].

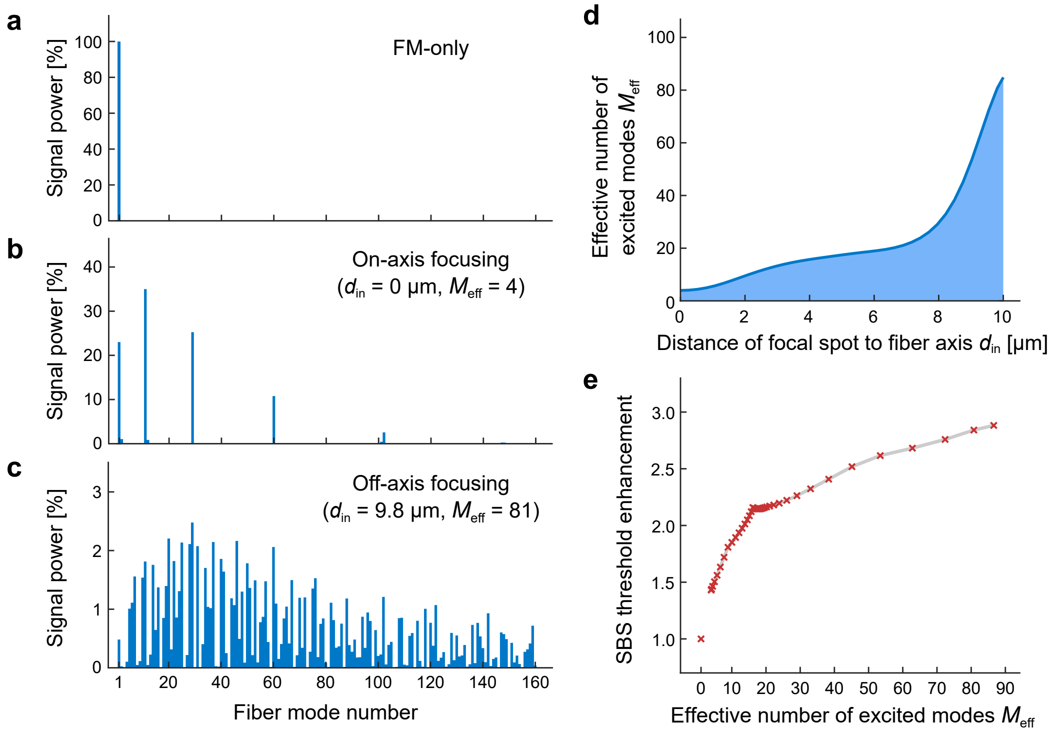

Fig. S6. Effective number of excited modes vs. SBS threshold. **a**–**c**, Excited signal mode contents at $\lambda = 1064$ nm for FM-only excitation (**a**), on-axis input focusing (**b**), and off-axis input focusing (**c**, near core edge) for a 50-meter-long 20-μm-core fiber, showing that the number of excited fiber modes increases from condition **a** to **c**. Modes are ordered according to their propagation constants. **d**, Effective number of excited modes (M_{eff}) vs. distance of input focal spot to fiber axis (d_{in}). M_{eff} increases from 4 to 85 as input focus is moved away from fiber axis. **e**, SBS threshold enhancement is raised from 1.4 to 2.8 as M_{eff} increases from 4 to 85 with input focusing.

Due to the exponential growth of Stokes power via SBS, the fiber mode with the highest Brillouin gain will dominate the reflected beam profile above the SBS threshold. The launching condition of the input light to the fiber determines the signal mode content, which in turn selects the Stokes mode with the highest Brillouin gain. In Fig. S7, we use our multimode SBS theory to predict the dominant Stokes modes for various distances of the input focus to the fiber axis, d_{in}, for linearly polarized excitation. When d_{in} is small (0–3 μm), mostly radial modes (signal) are excited, providing higher Brillouin gain for radial modes (Stokes). More specifically, the nearly degenerate HE_{12} and EH_{12} modes dominate the backward Stokes in this range [see the left inset in Fig. S7]. For larger d_{in}'s, the signal includes more non-radial modes, and the Brillouin gain is higher for non-radial modes. For $d_{\text{in}} \approx 5$–$7\ \mu$m, the lowest-order non-radial modes HE_{21} and EH_{21} begin to dominate the reflected beam profile above

the SBS threshold [right inset in Fig. S7]. The theoretical predictions match very closely with the experimentally measured reflection profiles in both cases [Fig. S7]. The far-field intensity pattern of Stokes above the SBS threshold is primarily a coherent superposition of HE_{12} and EH_{12} modes for $d_{in} \approx 0$–3 μm, and of HE_{21} and EH_{21} modes for $d_{in} \approx 5$–7 μm. At $d_{in} \approx 5$ μm, the dominant modes in Stokes switches, leading to a change of slope in the SBS-threshold-enhancement curve [main panel of Fig. S7].

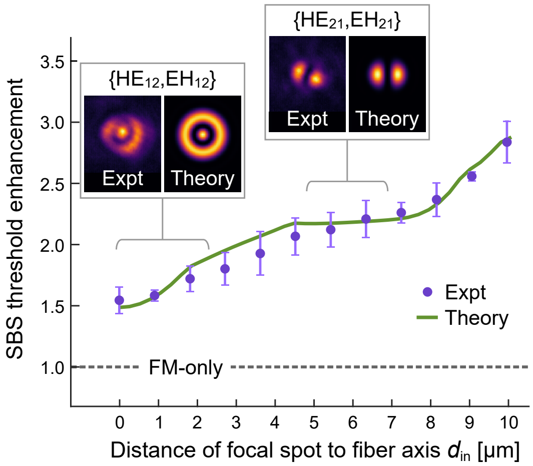

Fig. S7. Far-field intensity patterns of backward Stokes: theory vs. experiment. With a linearly polarized focused input, HE_{12} and EH_{12} modes experience the maximum Brillouin gain for the distance of focal spot to fiber axis $d_{in} \approx 0$–3 μm. For $d_{in} \approx 5$–7 μm, the dominant Stokes modes are HE_{21} and EH_{21}. These theoretical predictions are consistent with the experimentally measured Stokes profiles for respective d_{in}'s. Each profile is a coherent superposition of the HE and EH modes as predicted.

2.2 Modeling fiber imperfections

In Fig. 3a of the main text, the SBS threshold enhancement predicted by our multimode SBS theory agrees well with the experimental data. Since the experimental data are influenced by various imperfections in the fiber, such as mode-dependent loss, linear mode coupling, and polarization mixing, we have systematically included these effects in our theoretical model. Below, we provide details for experimental characterization and theoretical modeling of each of these effects individually, along with how they modify the SBS threshold enhancement.

Mode-dependent loss

As light propagates through the fiber, some of it leaks out of the core due to imperfect optical confinement. Such loss varies with fiber modes, typically the higher-order modes suffer more loss due to weaker confinement. To experimentally characterize the mode-dependent loss (MDL) in our fiber, we measure the transmittance as a function of the distance d_{in} of the input focus to the fiber axis. Without MDL, the fiber transmittance is expected to be unity for d_{in} less than the fiber core radius R (dashed blue curve in Fig. S8a), and to drop sharply for $d_{in} > R$. The experimentally measured transmittance (purple crosses) is less than unity for $d_{in} < R$ and continues to drop with increasing d_{in}. This is a manifestation of MDL with stronger loss for higher-order modes, because the number and order of excited HOMs increase with d_{in} [Fig. S3b–d]. To model it quantitatively, the loss coefficient of a mode is considered to vary quadratically with its azimuthal index, and the proportionality constant is a fitting parameter [7]. Our phenomenological model can be derived by *ab initio* methods [7, 8, 9] considering the fiber bending/twisting and the scattering due to disorder. Using this model, we calculate the fiber transmittance and vary the proportionality constant in the MDL model as the single fitting parameter to minimize the deviation from the measured transmittance for different d_{in}. The theoretical fit (solid green curve in Fig. S8a) agrees well with the experimental data, validating our model.

Once the MDL in our fiber is characterized experimentally, we include it in our theoretical prediction of the SBS threshold enhancement for different values of d_{in}. In Fig. S8b, the MDL lowers the SBS

threshold, because the effective number of excited modes is smaller. When the signal is focused at a larger d_{in}, more higher-order modes are excited, and they suffer stronger loss, leading to a larger drop of the SBS threshold in terms of transmitted power. This result suggests that reducing the MDL can lead to an even higher enhancement of the SBS threshold using multimode excitation.

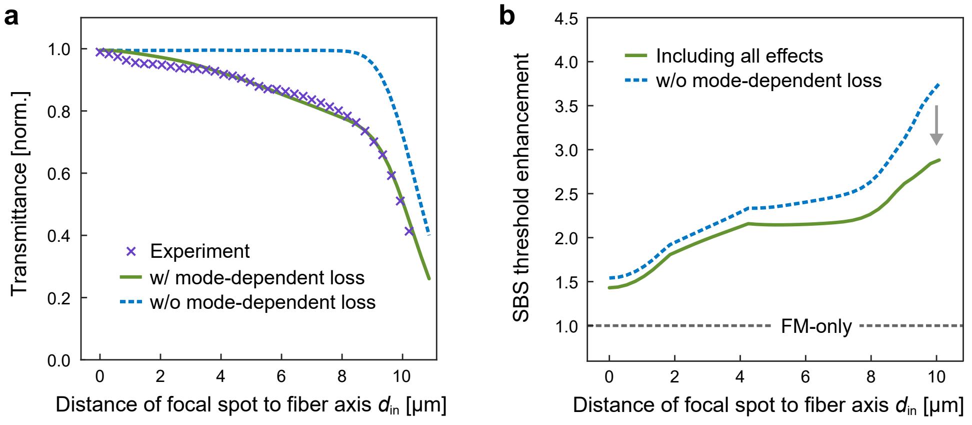

Fig. S8. Effect of mode-dependent loss on SBS threshold enhancement. **a**, Transmittance of light at $\lambda = 1064$ nm through a loosely coiled, 50-meter-long, 20-μm-core fiber vs. distance of input focal spot to fiber axis d_{in}, showing decrease in transmittance with increasing d_{in} due to stronger loss for higher order modes. Crosses: experimental data, solid/dashed curve: theoretical curve calculated with/without mode-dependent loss. **b**, Calculated SBS threshold enhancement over FM-only excitation with and without mode-dependent loss as a function of d_{in}. The loss causes a bigger drop of threshold enhancement at larger d_{in}, where more high-order modes are excited and suffer stronger loss. Both linear mode coupling and polarization mixing are included in the theoretical calculation.

Linear mode coupling

Without mode coupling, the mode content for a signal remains constant throughout the fiber. However, fiber imperfections and external perturbations cause linear mode coupling. This generically results in an increase in the effective number of excited modes, which raises the SBS threshold. Typically, the linear mode coupling is strongest for the neighboring modes and decreases with the increasing difference in modal propagation constants. We introduce the mode coupling with a banded random matrix shown in Fig. S9a. The matrix element T_{nl} dictates the power in mode n when unity power is input to mode l. We construct the coupling matrix $\mathbf{T} = \mathbf{I} + \gamma\,\mathbf{R}$, where $\mathbf{I}$ is an identity matrix, $\mathbf{R}$ is a banded random matrix with elements between 0 and 1, and γ determines the coupling strength. $\mathbf{T}$ is then normalized such that the sum of elements in each row is unity, which ensures power conservation.

Without linear mode coupling, when the input light is focused to the fiber axis ($d_{\text{in}} = 0$), only the FM and some radial HOMs are excited, resulting in a 1.2× enhancement of the SBS threshold. However, experimentally measured far-field intensity pattern of the transmitted light for $d_{\text{in}} = 0$ [Fig. 2f] reveals a small amount of non-radial HOMs, indicating the presence of weak linear mode coupling in the fiber. The observed SBS threshold enhancement is ∼1.5×, higher than the theoretical prediction without mode coupling. After selecting a banded random matrix $\mathbf{R}$ of bandwidth equal to 5 nondegenerate modes, we fit the value of γ such that the SBS threshold enhancement predicted with $\mathbf{T}$ matches the experimental value for $d_{\text{in}} = 0$. The fitting gives $\gamma = 0.03$, confirming weak linear mode coupling in our fiber. For all other values of d_{in}, we use the same coupling matrix $\mathbf{T}$ to correct for the excited mode contents, and then calculate the SBS thresholds.

The results for the SBS threshold enhancement with (solid curve) and without (dashed curve) the linear mode coupling are shown in Fig. S9b. As predicted, linear mode coupling generally increases the SBS threshold, and the increase is maximal for $d_{\text{in}} = 0$ and minimal for the largest d_{in} (= 10 μm). This is because for a large d_{in}, most modes are already excited at the fiber input, and linear mode coupling causes minimal changes in the mode content of the signal.

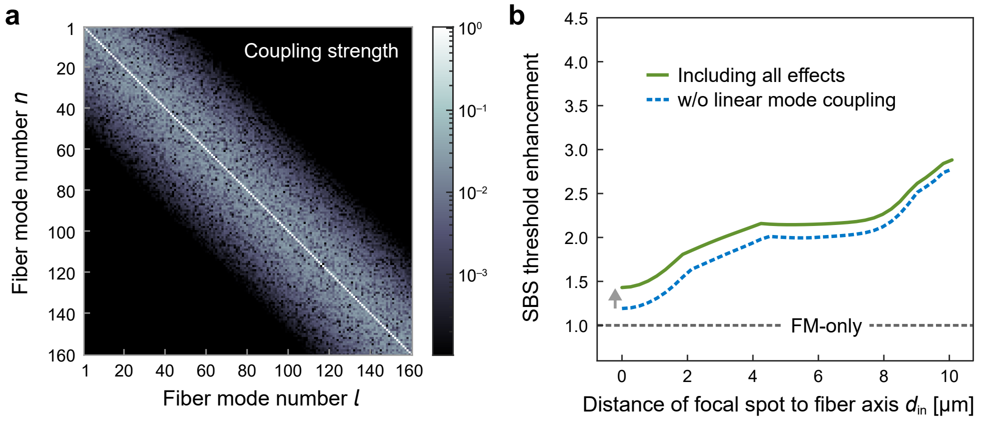

Fig. S9. Effect of linear mode coupling on SBS threshold enhancement. a, Banded random matrix $\mathbf{T}$ for simulating linear mode coupling in the MMF at $\lambda = 1064$ nm, obtained by fitting the experimental data in Fig. 3a of the main text. Coupling strength (plotted in log scale) is larger for modes with smaller difference in their propagation constants. **b**, Calculated SBS threshold enhancement over FM-only excitation with and without linear mode coupling as a function of input focusing distance from fiber axis, d_{in}. Linear mode coupling spreads signal power to more fiber modes and increases the SBS threshold enhancement. The effect is more dramatic at smaller d_{in}, since only a small number of fiber modes ($M_{\text{eff}} < 10$ for $d_{\text{in}} \sim 0$) are excited at the fiber input. Both mode-dependent loss and polarization mixing are included in the theoretical calculation.

Polarization mixing

Experimentally, linearly polarized light is coupled into a 50-meter-long MMF, and the output polarization state differs from the input one. This is attributed to two separate effects. Consider HOMs with non-zero azimuthal index in the same group, i.e., having identical intensity profiles but different polarization states. Their propagation constants are slightly different. When linearly polarized light excites a superposition of these modes at the fiber input, they will walk off upon propagation in the fiber, producing elliptically polarized light at fiber output. This process effectively causes a power division between two orthogonal polarizations and thus an enhanced SBS threshold for HOMs. We account for this effect in our model by utilizing vector modes instead of linearly polarized modes. In a perfect fiber, such an effect is absent for radially symmetric FM and HOMs. However, fiber imperfections and bending or twisting introduce weak birefringence and polarization mixing, even for the FM. Previous works have shown that, in a single-mode fiber with complete polarization scrambling by imperfection-induced weak birefringence, 1/3 of the input power couples to the FM with polarization orthogonal to the input [10].

To verify that these two effects are present in our fiber, we use a linear polarizer to characterize the output polarization state. With linearly polarized input, we measure the output intensity while rotating the linear polarizer. The ratio of minimum power P_{min} to maximum power P_{max} is plotted against the distance of the input focal spot to the fiber axis, d_{in}. We theoretically predict this dependence by combining the aforementioned two depolarization effects. Figure S10a shows that the theoretical prediction matches closely with the experimental data. At $d_{\text{in}} = 0$, $P_{\text{min}}/P_{\text{max}} \neq 0$, because the weak birefringence causes depolarization of the FM and a few radial HOMs that are excited. At a larger d_{in}, depolarization is stronger, as a large number of non-radial HOMs are excited.

The polarization mixing reduces the gain for SBS due to power division into two orthogonal polarizations. As a result, the SBS threshold for FM-only excitation is increased. This leads to a lower enhancement of the SBS threshold by multimode excitation, as the enhancement is given by the ratio of the multimode SBS threshold to the FM-only threshold. Figure S10b shows the threshold enhancement with (solid) and without (dashed) polarization mixing. Once the FM-only threshold is corrected for the depolarization effect, a lower enhancement of the SBS threshold reduces the slope of threshold enhancement with d_{in}.

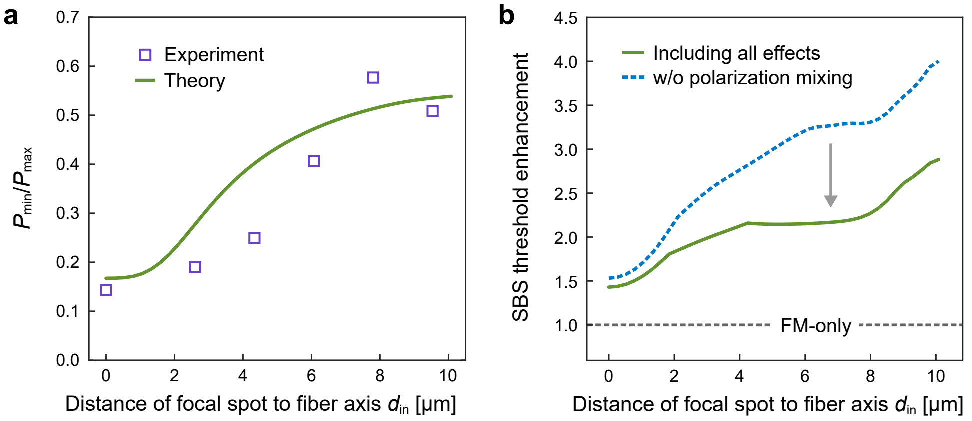

Fig. S10. Effect of polarization mixing on SBS threshold enhancement. **a**, Average ratio of minimum to maximum transmitted power through a linear polarizer at MMF output $P_{\mathrm{min}}/P_{\mathrm{max}}$, when linearly polarized light at $\lambda = 1064$ nm is focused to the fiber input facet at a distance d_{in} from the core center. Squares: experimental data, solid curve: theoretical estimation. **b**, Calculated SBS threshold enhancement with and without polarization mixing as a function of d_{in}. The slope is reduced by polarization mixing, which raises the SBS threshold for FM-only excitation. Both mode-dependent loss and linear mode coupling are included in the theoretical calculation.

2.3 Optimization of SBS suppression

Optimized mode contents

To gain insight into how an optimized input wavefront enhances the SBS threshold in a multimode fiber, we numerically simulate our wavefront shaping experiment. The same configuration and number of SLM macropixels are set up for phase modulation of a Gaussian beam similar to the CW experiment at $\lambda = 1064$ nm. For each SLM phase pattern, the SBS threshold is predicted by our multimode SBS theory including mode-dependent loss, linear mode coupling, and polarization mixing. The predicted threshold is used as the objective function for optimizing the phase modulation. Figure S11a shows an example of the optimization process starting with a random phase pattern. By optimizing the phase of the macropixels one by one, the threshold enhancement increases from 2.6 to 3.5 and then saturates after two to three rounds of optimization of all macropixels. This is in good agreement with the experimental result in Fig. 4b of the main text, and the optimized mode content is shown in Fig. S11b. We repeat the optimization process with different initial phase patterns and present two more examples in Fig. S11b. While the mode contents are different, they all feature a few widely spaced groups of modes. Instead of spreading input power into all modes, the optimization leads to a selective combination of modes that effectively utilizes the inhomogeneous intermodal and intramodal coupling strengths to maximize the SBS threshold.

Figures S11b and 4c reveal that different initial phase patterns and/or different sequences of macropixels for optimization will reach varying mode contents finally, but the corresponding values of SBS threshold enhancement are all around 3.3–3.5 [Fig. 4d]. To quantitatively evaluate how different the optimized mode contents are, we perform 50 optimizations with different initial phase patterns, and calculate the correlation between each pair of optimized mode contents $\{P_l(j)\}$, where l is the mode index, and j denotes the jth optimization. The Pearson correlation coefficient has a mean value of 0.87 and a standard deviation of 0.07. Thus most optimization processes find similar mode contents from different initial phases. However, the minimum correlation coefficient is merely 0.57. and the mode contents for the least correlated pair are shown in lower panels of Fig. S11b. Nevertheless, the corresponding SBS threshold enhancements are both 3.4, indicating different combinations of excited modes can reach comparable SBS thresholds that are notably higher than the random phase patterns. All optimizations take the same strategy to maximize the SBS threshold: exciting widely spaced groups of modes.

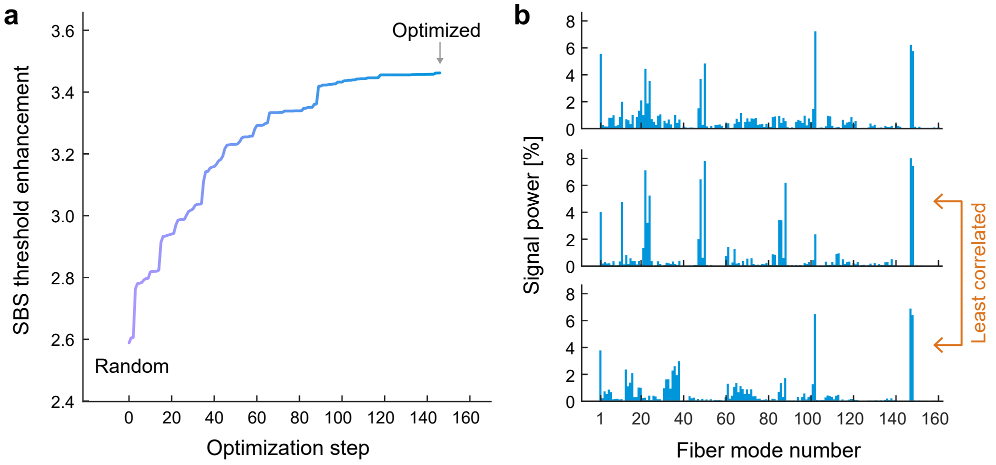

Fig. S11. Simulated optimization of SBS threshold enhancement. **a**, SBS threshold enhancement over FM-only excitation is recorded after optimizing the phase of each macropixel. The optimization starts with a random phase pattern of 8×8 macropixels, and the corresponding SBS threshold enhancement is $\sim$2.6. After two to three rounds of optimization of all macropixels, the threshold enhancement is saturated to $\sim$3.5. **b**, Three examples of optimized mode contents feature widely spaced groups of modes. Lower two panels are the pair with the least correlated mode contents among 50 optimized ones with different initial random phase patterns and different sequences of macropixel optimization.

Comparison of SBS suppression schemes

Figure S12 compares all the approaches employed in this work to realize multimode excitation for SBS suppression. First, tight focusing of input light to the fiber core center excites HOMs and increases the SBS threshold over FM-only excitation. Moving the input focus away from fiber axis further increases the effective number of excited modes M_{eff} from 4 to 85 (out of 160 modes), leading to a monotonic rise of SBS threshold enhancement up to 2.8. Next, random phase modulation of the input wavefront of linearly polarized light effectively excites 40–60 modes, resulting in SBS threshold enhancement of 2.3–2.7. Finally, wavefront optimization further pushes the threshold enhancement to 3.3–3.5. However, the effective number of modes is lower, $M_{\text{eff}} \sim 30$, illustrating the most efficient way of mitigating SBS is selective mode excitation, not uniform excitation.

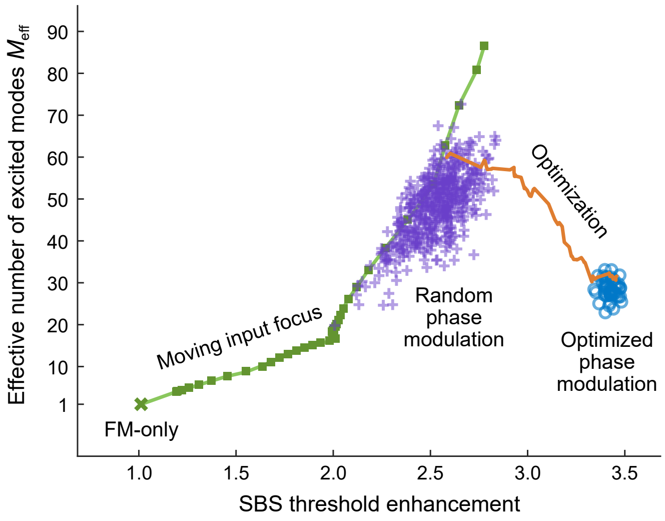

Fig. S12. SBS threshold enhancement vs. effective number of excited modes. SBS threshold increases by changing the excitation condition from FM-only (green $\times$) to multimode excitation with a tightly focused input beam (green ■). Moving the input focus from fiber core center to edge increases threshold up to 2.8. Multimode excitation by random phase modulation (purple $+$) leads to a SBS threshold enhancement of 2.3–2.7. Both cases display a positive correlation between the SBS threshold enhancement and the effective number of excited modes in the fiber. However, optimization of input phase modulation results in selective modal excitation (blue ◯) and a reduction in the effective number of excited modes for further enhancement of the SBS threshold.

To maximize the threshold, full control of input field amplitude, phase, and polarization in every fiber mode is needed. In the current experiments, the incident beam on the SLM features a Gaussian profile of its field amplitude. The SLM modulates only the phase front without changing the amplitude profile. Since the SLM plane corresponds to the far field of the fiber proximal facet, higher intensity of the incident beam near the center of its active region promotes the lower-order modes in the fiber, thus reducing the effective number of excited modes and the SBS threshold. With both amplitude and phase modulation of an input wavefront, we can enhance the HOM contents and the SBS threshold. Our multimode SBS theory predicts a further increase of the SBS threshold enhancement to 4.5 or more upon both amplitude and phase optimization, surpassing the maximum threshold enhancement of 3.5 achieved by phase-only optimization.

2.4 Output focusing efficiency

In our experiment, and simulation shown below, the efficiency of focusing light through a multimode fiber is given by the ratio of power within the focal spot to the total transmitted power. The focal spot diameter is equal to twice the full width at half maximum of the intensity. Experimentally, the focusing efficiency is $\sim$0.7 with phase-only optimization of the input wavefront [Fig. 5 in the main text]. To find the maximum focusing efficiency possible with phase-only modulation of a single polarization, we numerically simulate the output focusing through a multimode fiber [Fig. S13]. We utilize time-reversal symmetry, which is equivalent to phase conjugation for a continuous wave. A diffraction-limited CW source is placed close to the fiber distal facet and propagates through a 50-meter-long fiber to the proximal end. Since many modes are excited, their interference forms a speckle pattern, which is Fourier transformed to the SLM plane. To simulate phase-only modulation by the SLM, we apply a Gaussian profile to the field amplitude. Then we phase-conjugate the field and perform an inverse Fourier transform to obtain the field coupled to the fiber. Finally, we calculate the field transmitted through the fiber and reach the focal plane to compute the focusing efficiency.

Figure S13 (solid curve) shows the (azimuthally averaged) focusing efficiency as a function of the distance of the focal spot to the fiber axis. In our simulation, we ignore mode-dependent loss, linear mode coupling, and polarization mixing in the MMF, and use much smaller macropixels (and thus a smoother phase pattern) than in the experiment. With phase-only modulation of the input wavefront, the focusing efficiency is $\sim$0.8, and fluctuates slightly with the focus position. This result confirms that the focusing efficiency achieved experimentally is close to the theoretical limit. For comparison, the focusing efficiency with both amplitude and phase modulations is near unity [dashed curve in Fig. S13].

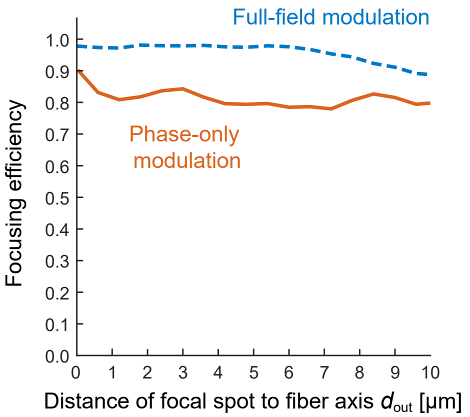

Fig. S13. Output focusing efficiency by input wavefront shaping. Compared to both amplitude and phase modulation of input wavefront, the efficiency of focusing light through a multimode fiber decreases from almost 1 to around 0.8 by phase-only modulation. The axial distance of the focal plane from the fiber output facet is 20 μm. Focusing efficiency varies slightly with the distance of the focal spot to the fiber axis, d_{out}, within the field of view.

Supplementary References

[1] R. Harrison, D. Yu, W. Lu, and P. Ripley, "Chaotic stimulated Brillouin scattering: theory and experiment," *Physica D: Nonlinear Phenomena*, vol. 86, no. 1-2, pp. 182–188, 1995.

[2] Y. Panbhiharwala, A. V. Harish, D. Venkitesh, J. Nilsson, and B. Srinivasan, "Investigation of temporal dynamics due to stimulated Brillouin scattering using statistical correlation in a narrow-linewidth cw high power fiber amplifier," *Optics Express*, vol. 26, no. 25, pp. 33 409–33 417, 2018.

[3] I. M. Vellekoop and A. Mosk, "Phase control algorithms for focusing light through turbid media," *Optics Communications*, vol. 281, no. 11, pp. 3071–3080, 2008.

[4] H. Yılmaz, W. L. Vos, and A. P. Mosk, "Optimal control of light propagation through multiple-scattering media in the presence of noise," *Biomedical Optics Express*, vol. 4, no. 9, pp. 1759–1768, 2013.

[5] D. B. Conkey, A. N. Brown, A. M. Caravaca-Aguirre, and R. Piestun, "Genetic algorithm optimization for focusing through turbid media in noisy environments," *Optics Express*, vol. 20, no. 5, pp. 4840–4849, 2012.

[6] Z. Bai *et al.*, "Narrow-linewidth laser linewidth measurement technology," *Frontiers in Physics*, vol. 9, 2021, ISSN: 2296-424X.

[7] A. R. Mickelson and M. Eriksrud, "Mode-dependent attenuation in optical fibers," *Journal of the Optical Society of America*, vol. 73, no. 10, pp. 1282–1290, 1983.

[8] R. Olshansky and D. Nolan, "Mode-dependent attenuation of optical fibers: Excess loss," *Applied Optics*, vol. 15, no. 4, pp. 1045–1047, 1976.

[9] K.-P. Ho, "Exact model for mode-dependent gains and losses in multimode fiber," *Journal of Lightwave Technology*, vol. 30, no. 23, pp. 3603–3609, 2012.

[10] M. O. Van Deventer and A. J. Boot, "Polarization properties of stimulated Brillouin scattering in single-mode fibers," *Journal of Lightwave Technology*, vol. 12, no. 4, pp. 585–590, 1994.
